# Assessment of F/HN-pseudotyped lentiviral vector following intravenous delivery to mice

**DOI:** 10.1038/s41434-026-00623-3

**Published:** 2026-06-18

**Authors:** Robyn V. Bell, Nikhil B. Faulkner, Anthony Sinadinos, Cuixiang Meng, Emily Castells, Mariana A. Viegas, Stephen C. Hyde, Deborah R. Gill, Eric WFW Alton, Uta Griesenbach

**Affiliations:** 1 National Heart and Lung Institute, Imperial College London, London, UK; 2 UK Respiratory Gene Therapy Consortium, London, UK; 3 Gene Medicine Research Group, Radcliffe Department of Medicine (NDCLS), University of Oxford, Oxford, UK

**Keywords:** Haematological diseases, Gene expression

## Abstract

In pursuit of a gene transfer agent with efficient pulmonary transduction, the UK Respiratory Gene Therapy Consortium has developed a lentiviral vector pseudotyped with the envelope proteins, F and HN from Sendai virus (rSIV.F/HN). In contrast to other viral vectors, pulmonary rSIV.F/HN delivery achieves sustained gene expression ( ~ 2 years in mice) in the lungs and systemic circulation following a single dose. Here, we investigate the application of the rSIV.F/HN vector-platform for wider indications, including systemic disorders that require serum expression of therapeutic proteins. To assess the potential for rSIV.F/HN to produce systemic proteins, intravenous vector delivery was characterised and compared against intrapulmonary administration, achieved via ‘nasal sniffing’. Both delivery routes achieved sustained (at least 1 year) systemic expression of the secreted reporter protein Gaussia luciferase. Systemic rSIV.F/HN delivery resulted in widespread protein expression across multiple organs, accompanied by the generation of significant anti-vector neutralising antibodies limiting vector readministration. Conversely, localised airway transduction was observed following pulmonary administration, which we have previously shown is not an impediment to efficient vector readministration. These data support intrapulmonary rSIV.F/HN delivery for systemic protein production, with sustained high-level transgene expression and feasible readministration.

## Introduction

Lentiviral vectors are highly effective gene transfer agents (GTAs) used to restore expression of deficient proteins in target cells. In pursuit of a GTA that achieves efficient pulmonary transduction, lentiviral vectors have been pseudotyped with envelope proteins from a range of lung pathogens, due to the latter’s efficiency at transducing the airway epithelium via the apical surface. These include lentiviral vectors pseudotyped with baculovirus, filovirus, influenza and paramyxovirus envelope proteins [1–4]. Given the high, but transient, transduction of lung epithelium using a Sendai virus-derived vector across many tissues, the UK Respiratory Gene Therapy Consortium developed a lentiviral vector (simian immunodeficiency virus (SIV)) pseudotyped with the envelope proteins, F and HN from Sendai virus (recombinant (r)SIV.F/HN) [5]. Proof-of-concept was generated in mice, demonstrating that rSIV.F/HN vectors can efficiently transduce multiple cell types in the lung in vivo [5–7]. Efficient and stable gene expression (approximately 2 years in mice) was observed after a single dose, with retained efficacy after repeated topical pulmonary administration [6]. In arguably more relevant models, including human and sheep ex vivo precision-cut lung slices, human nasal brushings and air-liquid interface cultures, persistent gene expression was again demonstrated [6, 7].

rSIV.F/HN has initially been assessed for the treatment of cystic fibrosis (CF) lung disease. The encouraging data generated catalysed the licensing of this platform technology expressing the cystic fibrosis transmembrane conductance regulator (CFTR) protein by Boehringer Ingelheim, and a first-in-human trial in CF subjects started at the end of 2024. In addition to the expression of membrane-bound proteins, such as CFTR, rSIV.F/HN has been utilised for the generation of both secreted proteins and antibodies within the lungs [8–10].

Broadening the application of lung gene transfer further, a strategy to use the respiratory epithelium as a factory for secretion of proteins to treat systemic diseases was explored. Proof-of-concept that the lungs could act as an efficient factory for the production and secretion of interleukin-10 into the circulation was demonstrated using recombinant Sendai vectors [11]. With the superior expression profile and reduced immunogenicity of rSIV.F/HN vectors, Paul-Smith et al. [8] investigated using the murine lungs as factories for systemic production using secreted Gaussia luciferase and the blood-clotting factor VIII as exemplars. Stable expression of the reporter protein was detectable in the blood for at least 12 months, with FVIII levels approaching therapeutic levels ( ~ 1% normal plasma levels) required by patients.

The majority of clinical trials that assess gene therapy treatments for systemic congenital disorders make use of an intravenous delivery route. This includes systemic AAV-based gene therapy for haemophilia A and B, which has demonstrated significant amelioration of bleeding phenotype in clinical trials [12], maintained expression of FIX in haemophilia B patients for >8 years following a single AAV dose [13] and recently led to the licensing of Roctavian and Hemgenix for haemophilia A and B, respectively. However, AAV vectors are unlikely to achieve sustained expression of therapeutic levels of protein due to their vector genomes remaining largely episomal, and thus repeated administration will be required, particularly in pediatric patients. Haemophilia B patients who received intravenous infusions of AAV2-FIX demonstrated persistent, high-titre neutralising antibodies (NAbs) against AAV2 and cross-reactive NAbs against other AAV serotypes (AAV5 and AAV8) for up to 15 years post-administration [14]. Similarly, all patients (*n* = 3) administered intravenous AAV5-FIX during the phase 2b clinical trial of Hemgenix recorded maximal titres of anti-AAV5 NAbs during the three-year follow-up [15]. The long-term persistence of NAbs following intravenous infusion of AAV vectors suggests that repeat intravascular administration to patients is not currently feasible for AAV vectors [16].

Following intranasal administration, rSIV.F/HN transduces a range of pulmonary epithelial cells, including terminally differentiated airway epithelial cells as well as alveolar type 1 and type 2 cells [7], the latter being progenitor cells with self-renewal capacity. Furthermore, rSIV.F/HN can be repeatedly administered to the lungs of mice without loss of transduction efficiency [6]. Thus, for the treatment of some chronic disorders, rSIV.F/HN may represent an attractive alternative to systemic delivery of AAV vectors. All previous studies investigating rSIV.F/HN have targeted the nasal or pulmonary epithelium via the ‘nasal sniffing’ route of administration. Intravenous vector administration for the delivery of transgenes encoding systemic proteins has not yet been investigated using this vector. We have, therefore, assessed the potential of the rSIV.F/HN vector for systemic protein production, comparing intravenous and intrapulmonary administration, the latter delivered through the standard ‘intranasal sniffing’ technique. Duration of expression and biodistribution were assessed for both routes, as well as the feasibility of efficient protein production following repeated intravenous administration.

## Methods

### Vector production and titration

Recombinant SIV.F/HN vector was produced by transient transfection of plasmids into HEK293T cells maintained in suspension and purification by anion exchange chromatography and tangential flow filtration, following previously detailed methodology [7]. Vectors were formulated in TSSM buffer (tromethamine 20 mM, NaCl 100 mM, sucrose 10 mg/mL, and mannitol 10 mg/mL). The HEK293T/17 cell line was utilised to allow vector production to be scaled up to 5 litre batches. The five plasmids required to produce third-generation SIV-based lentiviral vectors included packaging plasmids encoding SIV gag/pol and SIV rev, plasmids encoding the SeV envelope proteins Fct4 and SIVct+HN and the vector genome. Vector genome plasmids included codon-optimised, CpG-free cDNAs encoding Gaussia luciferase (GLux) and the firefly luciferase/EGFP fusion (EGFPLux). Vector genome encoding murine ADAMTS13 (a disintegrin and metalloproteinase with thrombospondin type 1 motif, 13), a secreted enzyme involved in blood clotting, served as a negative control vector as it was found previously to produce non-detectable levels of protein in sera when delivered to mice. To drive transgene expression, all vector genomes included our in-house CpG-free promoter/enhancer fusion (hCEF), consisting of the short human EF1α promoter coupled to the human CMV enhancer.

To determine the functional titre of the recombinant vector, the infectious activity was measured in an adherent HEK293T cell line following serial dilution of viral vector and extraction of genomic DNA using the QIAamp DNA Mini Kit (Qiagen, Crawley, UK). Vector genomes were quantified by Real Time Quantitative PCR (qPCR) using TaqMan Universal Mastermix (Life Technologies, Paisley, UK) and primers targeting the WPRE sequence (Forward: TGGCGTGGTGTGCACTGT; Reverse: CCCGGAAAGGAGCTGACA; Probe: 6FAM-TTGCTGACGCAACCCCCACTGG-TAMRA), comparing against a standard curve of plasmid DNA containing the WPRE sequence. Infectious titre in transduction units per ml (TU/ml) was calculated from the slope of the line of best-fit on a plot of WPRE copies per well and by accounting for the vector dilution.

### In vivo transduction

All animal procedures were performed in accordance with the UK Home Office Project and Personal licence regulations under the Animal Scientific Procedure Act (1986). C57BL/6 mice were purchased from Charles Rivers Laboratories (Margate, UK) and, unless otherwise stated, were 8–10 weeks of age at the beginning of studies. Mice were housed in individually ventilated cages with food and water *ad libitum* and with 12 h light/dark cycles. Sample size was selected based on previous experience using the rSIV.F/HN vector. All samples collected were included in the analysis. Mice were randomly allocated to the different treatment groups using a random number generator to avoid bias. Analysis was performed in a non-blinded manner. For intrapulmonary (IP) dosing, mice were anaesthetised with isoflurane (1 L/min oxygen and 3% isoflurane), then administered recombinant SIV vector (100 µl total volume/dose) by nasal sniffing. Briefly, anaesthetised mice were held upright with their mouth gently shut and liquid applied to the nostrils, which was voluntarily inhaled by the mouse into the lungs. To dose intravenously (IV), mice pre-warmed in a heating chamber, were secured in a restriction tunnel and vector (100 µl total volume/dose) injected into either of the lateral tail veins using a 25 gauge insulin needle. Control animals received TSSM (100 µl) by intrapulmonary or intravenous administration or were untransduced negative controls.

### Gaussia luciferase duration expression study

Vector encoding GLux (rSIV.F/HN-GLux) was delivered to wildtype mice to compare secretion of protein into the blood following intravenous or intrapulmonary vector administration. Mice were treated with increasing doses of vector (2.4e7, 7.6e7, 2.4e8 TU/mouse, *n* = 6 mice/group) or TSSM buffer via either delivery route. To measure serum levels of GLux in the same mouse over time, blood was repeatedly sampled through tail venepuncture (1, 2, 5, 12, and 24 weeks post-dosing) as described above.

For the preparation of serum, blood was collected without an anticoagulant, allowing clots to form at room temperature for at least 15 min. Sera were separated by centrifugation at 1500 × *g* for 10 minutes at 4 °C, followed by storage of aliquots at −80 °C. At 52 weeks post-dosing, mice were culled by overdose with pentobarbital, blood extracted by cardiac puncture and serum prepared as described above. Bronchoalveolar lavage fluid (BALF) was collected by securing a 22 gauge canula into the trachea and washing the lungs three times with a syringe containing 200 μl of PBS (−/−). BALF was centrifuged at 5000 × *g* for 10 minutes, supernatant removed, and then stored at -80 °C. GLux expression was quantified in serum and BALF (5 µl of sample/assay) using the Pierce Gaussia Luciferase Flash Assay Kit (Thermo Fisher Scientific, Waltham, Massachusetts, USA) following the supplier’s protocol. Coelenterazine substrate is oxidised by GLux enzyme, emitting luminescence of wavelength 470–600 nm, detected by the FLUOstar Omega plate reader (BMGLabtech, Ortenberg, Germany). GLux expression was expressed as relative light units (RLU).

### Vector biodistribution study

To investigate vector biodistribution, rSIV.F/HN-EGFPLux was delivered intravenously (*n* = 4 females and *n* = 4 males, 2.58e8 TU/mouse) and via the intrapulmonary route (*n* = 3 females and *n* = 3 males, 2.58e8 TU/mouse) to mice, alongside untransduced mice (*n* = 3 females and *n* = 2 males). In vivo bioluminescence imaging was performed 35 days post-dosing. Mice depilated under isoflurane anaesthetic one day previously received 150 mg/kg of VivoGlo Luciferin (Promega, Southampton, UK) by intraperitoneal injection. Luciferin is oxidised by firefly luciferase, producing luminescence detected at wavelength 562 nm, which 15 min after injection, was imaged using the In Vivo Imaging System (IVIS) Spectrum (Perkin Elmer, Waltham, Massachusetts, USA) with mice kept under isoflurane anaesthesia. Organs were subsequently dissected from each mouse (liver, lung, spleen, brain, kidney, heart and gonads) immediately after whole-body imaging and placed in petridishes for imaging.

Small amounts of each dissected organ (< 50 mg tissue) were homogenised in Reporter Lysis Buffer (Promega) for quantification of luciferase activity and protein analysis. Samples were loaded into Lysing Matrix D tubes (MP Biomedicals, Santa Ana, California, US) and processed using a Fast-Prep homogeniser (Thermo Fisher Scientific) at 4.0 meters/second for 45 s. The homogenate was transferred to a Qiashredder column (Qiagen) and centrifuged at 10,000 × *g* for 1 min. Columns were removed and the homogenate centrifuged for an additional 5 min. Firefly luciferase activity was measured in organ homogenates (20 µl of sample/assay) using the Bio-Glo Luciferase Assay System (Promega) following the supplier’s protocol. 5´-fluoroluciferin is oxidised by firefly luciferase, emitting luminescence detected at wavelength 562 nm, which is detected using the FLUOstar Omega plate reader. Total protein was determined using a DC Protein Assay Kit (BioRad, Hercules, California, US) according to manufacturer’s instructions and normalised bioluminescence signal expressed as relative light units (RLU)/mg total protein.

### Repeat intravenous administration study

Repeat administration was investigated by comparing transgene expression between mice receiving two doses of control vector (rSIV.F/HN-mADAMTS13, 1.42e8 TU/mouse) followed by a final dose of rSIV.F/HN-EGFPLux (1.52e8 TU/mouse, n = 6 mice) and mice that received two doses of TSSM buffer followed by a single dose of rSIV.F/HN-EGFPLux (1.52e8 TU/mouse, n = 6). Luciferase expression was quantified at the terminal time point only. Mice received doses (control vector or TSSM) at monthly intervals. One month following the final dose, mice underwent whole-body bioluminescence imaging using the IVIS Spectrum, as described above. Densitometry analysis of bioluminescence images was performed using ImageJ [17], taking area under curve (AUC) measurements as a readout of luciferase expression. Organs (as described above) were subsequently dissected from each mouse and imaged. Measurement of firefly luciferase activity in organ homogenate was performed as described above.

To measure levels of neutralising antibodies in mice that received one, two or three intravenous doses of rSIV.F/HN, blood was sampled from mice one month after each dose was given, prior to administration of the subsequent dose and serum was prepared as described above. Due to the technical difficulties of collecting sufficient blood whilst also dosing mice, only a subset of animals were sampled and those that received two and three vector doses were grouped together. Neutralising antibody measurements in mouse serum were performed by incubation of rSIV.F/HN-EGFP with 1 in 2 serial dilutions of serum from 1/50 up to 1/6400. Serum and vector were incubated for 30 minutes at 37 °C, then applied to HEK293T cells seeded in 96-well plates (3 000 cells/well). The percentage of transduced (GFP +) cells was assessed by flow cytometry 72 h later using standard methodology. The inverse serum dilution leading to 50% reduction of GFP + cells was taken as the neutralising titre (IC50).

### Statistical analysis

Statistical analyses were performed using GraphPad Prism 11 (GraphPad Software, La Jolla, California, USA). Gaussian distribution and lognormal distribution were assessed using the D’Agostino-Pearson omnibus normality test. Where lognormal distribution was established, data were transformed by taking the logarithm of each value. Datasets where Gaussian and lognormal distributions were established (*p* > 0.05) were analysed using parametric statistical tests, and data plotted as mean ± standard deviation (SD). Where data were not Gaussian or lognormally distributed, non-parametric tests were utilised, and data expressed as median + range. Unpaired *t*-test was used for two-group comparison, and for multi-group analysis, Kruskal-Wallis test followed by Dunn multiple comparison post-hoc test was performed. To analyse serum GLux expression over time, a linear mixed-effects model was utilised to account for both fixed and random effects in the experimental design. Delivery route, vector dose and time were included as fixed effects, along with all possible interactions. Individual mice were included as a random effect. To investigate tissue luciferase expression after vector administration, we employed a two-way mixed-effects ANOVA. The model included treatment route or treatment number and organ as fixed effects, along with their interaction. Individual mice were included as a random effect. Significant interactions were followed up with post-hoc pairwise comparisons using Tukey’s adjustment. The null hypothesis was rejected at *p* < 0.05.

## Results

### Systemic expression of Gaussia luciferase is stable for at least 12 months following a single dose

To investigate the suitability of rSIV.F/HN vector for treatment of systemic diseases, intravenous administration to wildtype mice was characterised and compared with intrapulmonary delivery. Across the 52-week study, three mice died prematurely from the intrapulmonary group and none from the intravenous group. Of these, one was found dead (intrapulmonary 2.4e7 TU/mouse group, 36 weeks post-transduction), one was culled due to an infected wound to the eye (intrapulmonary 7.6e7 TU/mouse group, 10 weeks post-transduction), and one was culled due to dermatitis caused by overgrooming (intrapulmonary TSSM group, 40 weeks post-transduction). Thus, 45/48 mice reached endpoint harvesting. GLux measurements from serum samples collected prior to the mice being culled were included in analyses.

**Fig. 1:**
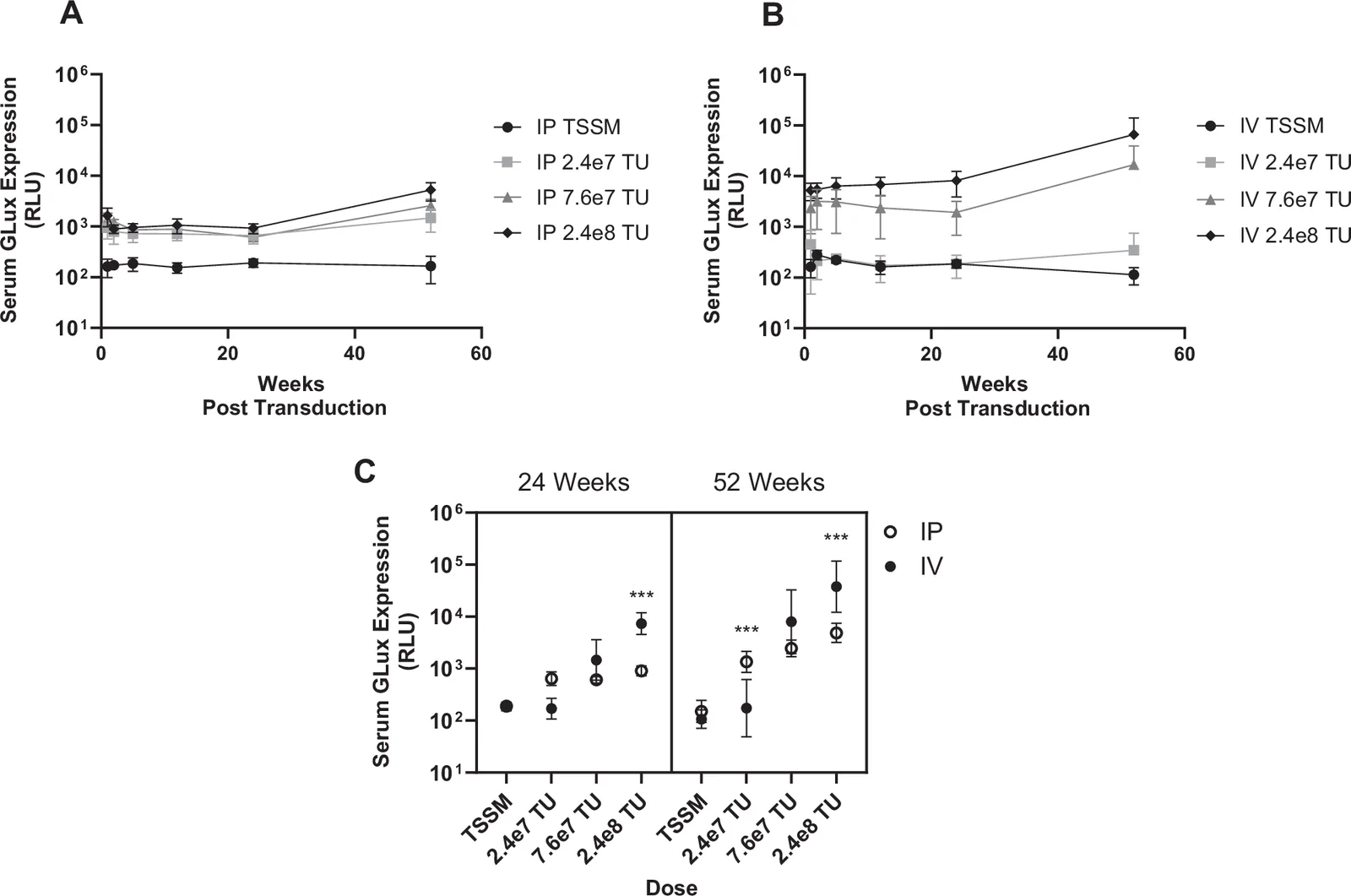
Serum Gaussia luciferase (GLux) expression over 12 months following a single dose of rSIV.F/HN. Wildtype C57BL/6 female mice received increasing doses of rSIV.F/HN-GLux (2.4e7, 7.6e7, 2.4e8 transduction units (TU)/mouse) while control mice received TSSM buffer (*n* = 5-6 mice/group). At regular intervals post-transduction (1, 2, 5, 12, 24, and 52 weeks) blood was sampled from mice, and GLux expression was quantified in serum, expressed as relative light units (RLU). Data expressed as mean ± standard deviation. **A** Mice delivered vector by intrapulmonary (IP) administration. **B** Mice delivered vector by intravenous (IV) administration. **C** GLux expression 24 weeks (experiment midpoint) and 52 weeks post-transduction (experiment endpoint) was compared between mice dosed by intrapulmonary (IP open circles) and intravenous (IV filled circles) administration for each treatment dose, expressed as relative light units (RLU). Data expressed as mean ± standard deviation. A linear mixed-effects model with dose, time, and delivery route as fixed effects and the individual mouse as a random effect. Post-hoc pairwise comparisons with Tukey’s adjustment *** = *p* < 0.001 when IP and IV are compared.

Systemic transgene expression was stable and persisted for the duration of the study (52 weeks) following a single intrapulmonary dose of rSIV.F/HN-GLux (Fig. 1A). All three treatment groups (2.4e7, 7.6e7 and 2.4e8 TU/mouse) resulted in GLux expression above background levels observed in control (TSSM) mice, at each time point sampled. Mice delivered intravenously rSIV.F/HN-GLux similarly showed persistent transgene expression for the duration of the experiment (Fig. 1B). However, serum levels of GLux above background were observed only in the two higher dose groups (7.6e7 and 2.4e8 TU/mouse), with the lowest dose resulting in minimal serum expression above background (TSSM) mice.

### Intravenous administration of rSIV.F/HN vector results in superior transduction efficiency at the highest treatment dose

To compare transduction efficiency between delivery methods, Fig. 1C shows GLux expression observed in each treatment group at the study midpoint and endpoint (24 and 52 weeks post-treatment, respectively). A linear mixed-effects model with dose, time, and delivery route as fixed effects and individual mouse as a random effect was performed. Dose and time were significant (*p* < 0.0001, respectively) main effects, as well as significant (*p* < 0.0001) interactions of dose × time and dose × delivery route, respectively. However, the delivery route was not determined to have a significant effect, and the interaction time × delivery route and dose x time x delivery route were also found to be not significant. Post-hoc analysis showed no significant difference between intrapulmonary and intravenous TSSM groups at both time points. Within the low dose group (2.4e7 TU/mouse), mice treated with vector by intrapulmonary administration demonstrated significantly (*p* < 0.001) higher serum GLux expression than intravenous mice at 52 weeks post-treatment, but not at the study midpoint (24 weeks). Whereas no significant difference was found between delivery routes in the medium dose groups at both time points. Finally, in the high dose cohort (2.4e8 TU/mouse), serum GLux levels were significantly (*p* < 0.001) higher following intravenous vector delivery at both 24 and 52 weeks post-treatment, respectively.

### Intravenous administration of rSIV.F/HN results in widespread biodistribution

**Fig. 2:**
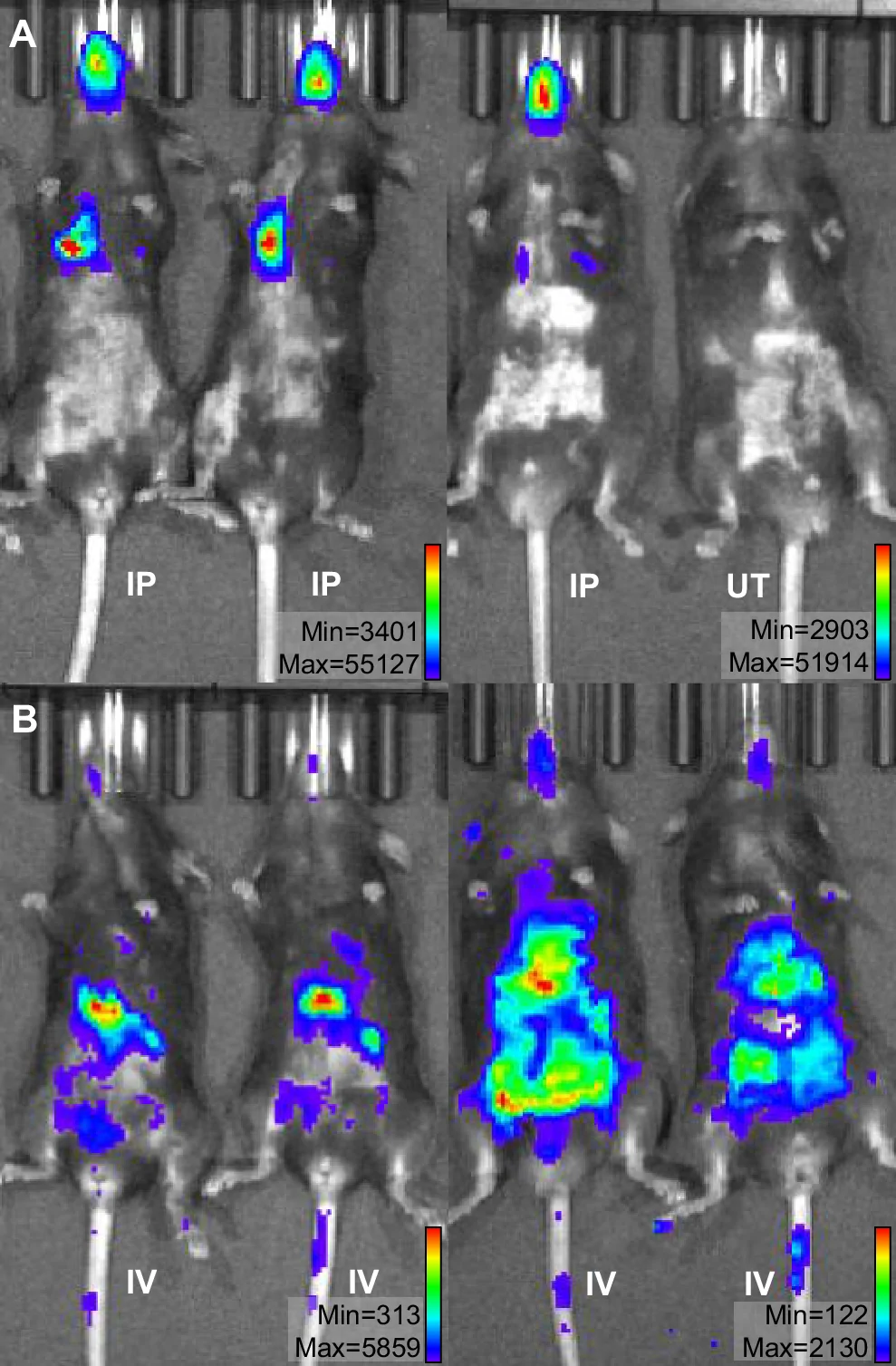
In vivo bioluminescence imaging following intrapulmonary and intravenous delivery of rSIV.F/HN-EGFPLux. Vector encoding the firefly luciferase and EGFP fusion (rSIV.F/HN-EGFPLux) was delivered to C57BL/6 wildtype mice by intrapulmonary (*n* = 3 females and *n* = 3 males, 2.58e8 TU/mouse) and intravenous administration (*n* = 4 females and *n* = 4 males, 2.58e8 TU/mouse) alongside untransduced mice (*n* = 3 females and *n* = 2 males). 35-days post-transduction, each mouse received 150 mg/kg of luciferin substrate by intraperitoneal injection, and 15-minutes later imaged using the IVIS Spectrum under isoflurane anaesthesia. Representative images of (**A**) 2 female mice and 1 male mouse delivered vector by intrapulmonary administration (IP) and 1 male untransduced mouse (UT), 3-min exposure per image, and (**B**) 2 female and 2 male mice delivered vector intravenously (IV), 5-min exposure per image. Min and Max represent the minimum and maximum signal, measured in counts, per image.

To assess biodistribution following either delivery route, rSIV.F/HN-EGFPLux was administered intravenously (*n* = 4 females and 4 males, 2.58e8 TU/mouse) and by intrapulmonary administration (n = 3 females and 3 males, 2.58e8 TU/mouse) to wildtype mice alongside untransduced mice (n = 3 females and 2 males). Figure 2 shows representative in vivo bioluminescence images taken 35-days post-dosing, with vector distribution patterns following intrapulmonary (Fig. 2A-IP) and intravenous (Fig. 2B-IV) administration shown alongside an untransduced mouse (Fig. 2A-UT). Bioluminescence signal was strongly localised to the nose and lungs in mice administered vector to the nasal cavity, however the expression pattern was more widespread when administered intravenously, with signal distributed across the lower thorax and abdomen, in the nose and in the tail. The untransduced mouse shown in Fig. 2A shows no evidence of bioluminescence signal.

Following in vivo imaging, seven different organs (lung, liver, kidney, heart, brain, gonads, spleen) were dissected from each mouse and imaged. Supplementary Fig. 1 shows representative images from an intrapulmonary dosed and an untransduced mouse (Supplementary Fig. 1A) as well as two mice administered vector intravenously (Supplementary Fig. 1B). Using a 5-minute exposure to capture each image, a saturation in bioluminescence signal was measured in the lungs of mice that received vector by intrapulmonary administration. The data show that even following prolonged photon detection, there is no evidence of luciferase expression in organs other than the lungs. Alternatively, positive bioluminescence was observed in the lungs, liver, kidney, gonads (testes in the representative image) and spleen of organs dissected from intravenously dosed mice.

**Fig. 3:**
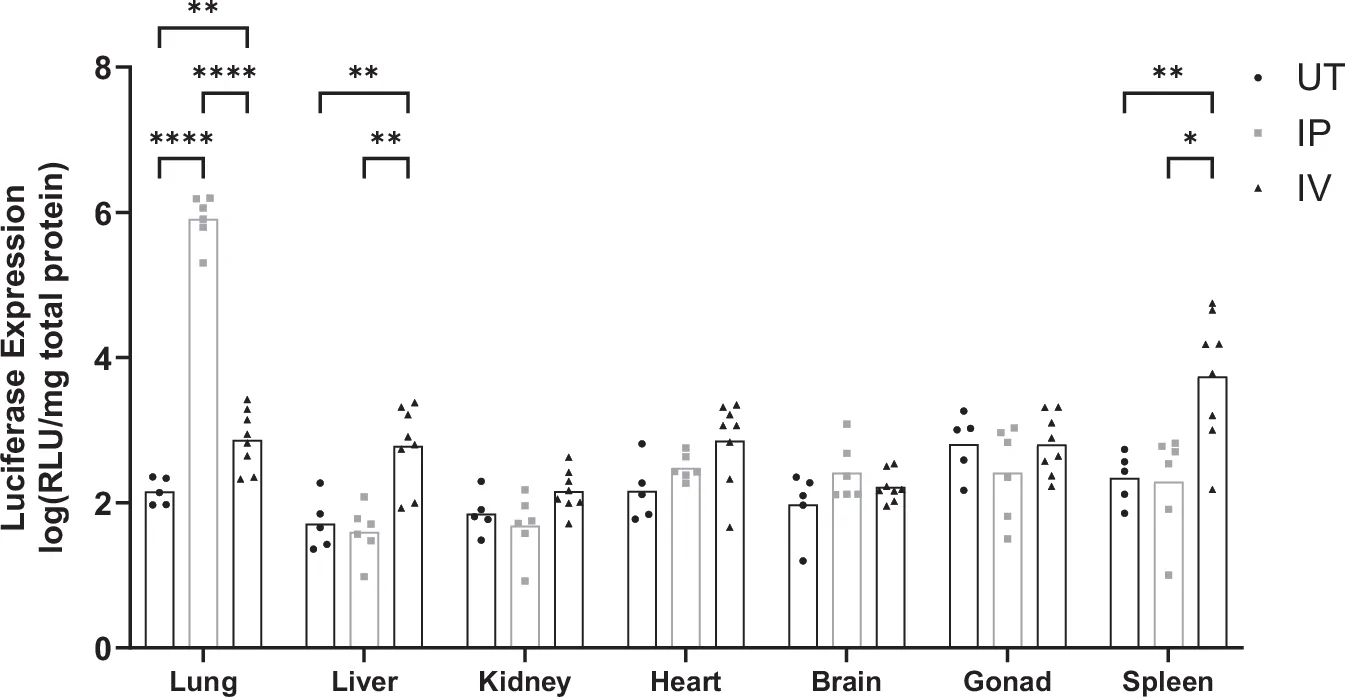
Biodistribution of rSIV.F/HN vector following intrapulmonary and intravenous administration. Vector encoding the firefly luciferase and EGFP fusion (rSIV.F/HN-EGFPLux) was delivered to C57BL/6 wildtype mice by intrapulmonary (*n* = 3 females and *n* = 3 males, 2.58e8 TU/mouse) and intravenous administration (*n* = 4 females and *n* = 4 males, 2.58e8 TU/mouse) alongside untransduced mice (*n* = 3 females and *n* = 2 males). 35-days post-transduction, mice were culled and luciferase activity quantified in vitro and normalised for total protein, expressed as relative light units (RLU)/mg total protein. Data has been log transformed; each bar represents mean luciferase expression per organ, and each shape represents a single mouse. Data from untransduced (UT black circles), intrapulmonary (IP light grey squares) and intravenous mice (IV dark grey triangles). Two-way mixed-effects ANOVA with fixed effects organ and treatment and the individual mouse as a random effect. Post-hoc pairwise comparisons with Tukey’s adjustment * = *p* < 0.05, ** = *p* < 0.01, *** = *p* < 0.001 when treatment conditions were compared.

To quantify biodistribution more accurately, organ homogenate was prepared from each of the seven organs and luciferase activity was measured. Figure 3 compares luciferase bioluminescence between mice delivered vector by intravenous and intrapulmonary administration to untransduced mice. Intravenous administration of vector resulted in a broader and overall weaker expression pattern compared with the strong, localised pulmonary signal observed following intrapulmonary delivery. A two-way mixed-effects ANOVA with fixed effects of organ and treatment and individual mouse as a random effect revealed significant (*p* < 0.0001 and *p* < 0.01) main effects of organ and treatment, respectively, as well as a significant (*p* < 0.0001) organ and treatment interaction. Post-hoc analysis showed significant (*p* < 0.01) expression above background untransduced levels was detected in the lungs, liver and spleen, respectively, following intravenous administration and significant (*p* < 0.01 and *p* < 0.05) expression above intrapulmonary mice in the liver and spleen, respectively. Following intrapulmonary administration, significant (*p* < 0.0001) luciferase expression above untransduced and intravenous mice was identified in the lungs, respectively. The biodistribution patterns characterised by in vivo imaging, organ imaging and in vitro luciferase quantification were consistent, demonstrating broader biodistribution following intravenous delivery compared to highly localised intrapulmonary delivery of rSIV.F/HN to mice.

### Transduction efficiency is reduced following repeat intravenous administration

**Fig. 4:**
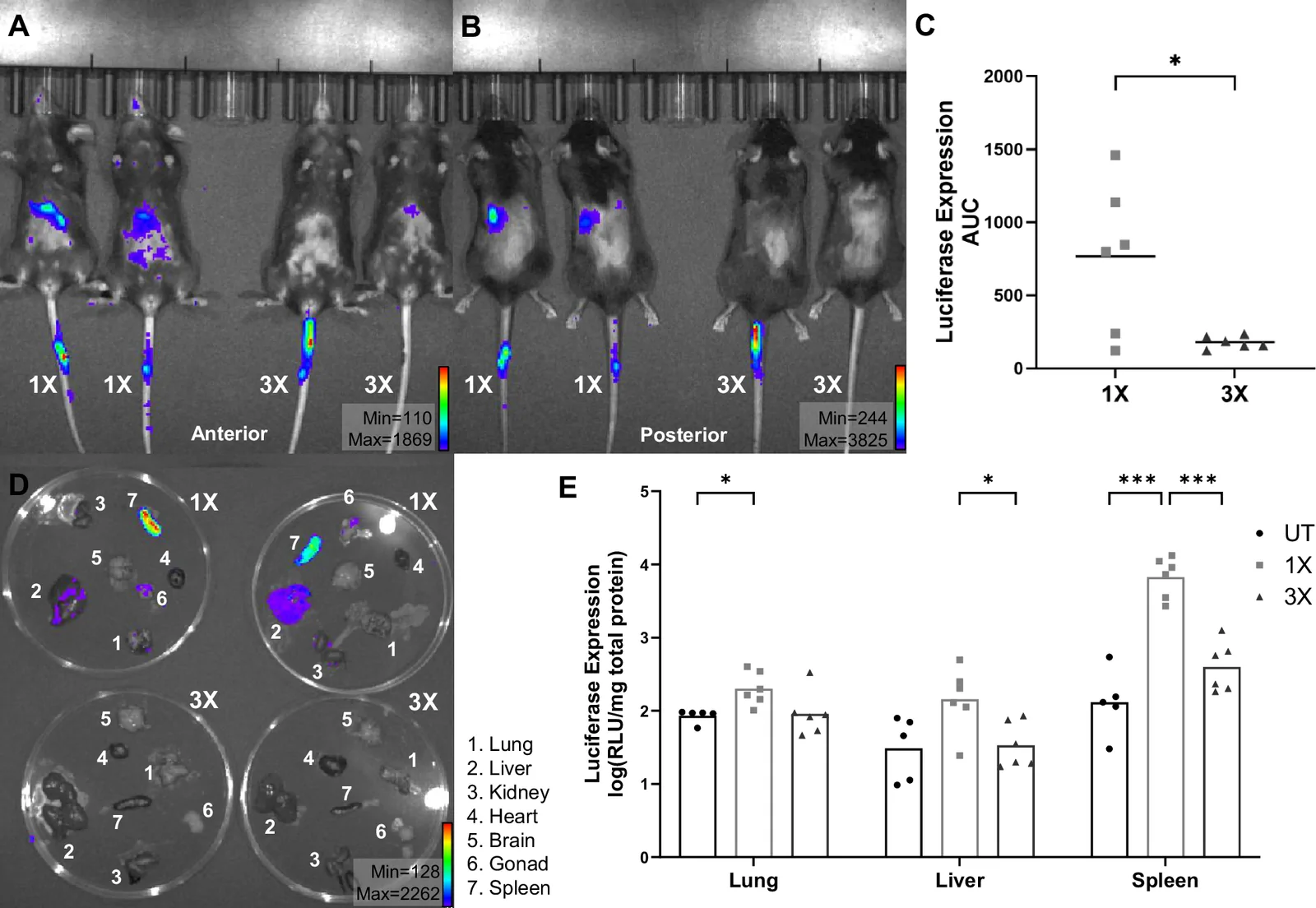
Bioluminescence imaging and analysis of organ expression following repeat intravenous administration of rSIV.F/HN. Wildtype C57BL/6 female mice were split into two groups at random. The single dose group (1X) received two doses of TSSM buffer followed by a single dose of rSIV.F/HN-EGFPLux (1.52e8 TU/mouse, *n* = 6 mice). The triple dose group (3X) received two doses of control vector (rSIV.F/HN-mADAMTS13, 1.42e8 TU/mouse) followed by a final dose of rSIV.F/HN-EGFPLux (1.52e8 TU/mouse, *n* = 6 mice). Mice received intravenous doses (vector or TSSM) at monthly intervals, and bioluminescence was quantified at the terminal endpoint only. One month following the final dose, each mouse received 150 mg/kg of luciferin substrate by intraperitoneal injection, and 15-minutes later, imaged using the IVIS Spectrum under isoflurane anaesthesia. Representative images showing (**A**) the anterior view and (**B**) the posterior view of two mice administered a single dose, and two mice administered three vector doses, 3-minute exposure per image. (**C**) Analysis of anterior bioluminescence image data, showing area under curve (AUC) measurements as a readout of luciferase expression. AUC values shown for mice that received a single dose (1X light grey squares) and three doses (3X dark grey triangles). Horizontal bars represent mean AUC values, each shape represents a single mouse. Significant reduction in overall signal between mice that received 1 dose and 3 doses of rSIV.F/HN (* = *p* < 0.05, unpaired *t*-test *n* = 6 mice/group). **D** Organs were subsequently dissected from each mouse- (1) lung, (2) liver, (3) kidney, (4) heart, (5) brain, (6) gonads, and (7) spleen and imaged, 5-minute exposure per image. Min and Max represent the minimum and maximum signal, measured in counts, per image. Representative images showing bioluminescence imaging of two mice administered a single dose of vector (1X) and two mice administered three doses (3X). **E** Luciferase activity was quantified in vitro and normalised for total protein, expressed as relative light units (RLU)/mg total protein. Previous data measured in untransduced mice (UT, black circles *n* = 5 mice) are shown as a comparison against mice that received a single dose (1X light grey squares) and three doses (3X dark grey triangles). Data have been log transformed, each bar represents the mean luciferase expression per organ, and each shape represents a single mouse. Two-way mixed-effects ANOVA with fixed effects of organ and dose number, and the individual mouse as a random effect. Post-hoc pairwise comparisons with Tukey’s adjustment * = *p* < 0.05, *** = *p* < 0.001 when treatment groups were compared.

To investigate if repeat administration of rSIV.F/HN following intravenous delivery is feasible, mice were treated with three doses of rSIV.F/HN vector at monthly intervals, ensuring the final vector encoded a different transgene from the previous two doses to differentiate anti-vector immune responses from possible anti-transgene immune responses. Luciferase expression was quantified at the terminal time point only, and transgene expression compared between mice receiving two doses of control vector expressing an irrelevant transgene, rSIV.F/HN-mADAMTS13, followed by a final dose of rSIV.F/HN-EGFPLux (3 doses (3X)) and mice that received two doses of TSSM buffer followed by a single dose of rSIV.F/HN-EGFPLux (1 dose (1X)). Figure 4A, B show representative in vivo bioluminescence images of the anterior and posterior views of the mice, respectively, using a 3-minute exposure for each image. In both images, there is a reduction in signal from the lower thorax and abdomen in the 3-dose group compared with mice receiving only one dose. To quantify the difference in transgene expression, analysis of anterior whole-body bioluminescence images was performed and AUC measurements were taken as a readout of luciferase expression (Fig. 4C). A statistically significant (*p* < 0.05) reduction in AUC measurements was found between mice that received 1 dose and 3 doses of rSIV.F/HN. The mean AUC value calculated was 77% lower in the mice receiving three doses compared to a single dose (mean ± standard deviation = 1 dose (767.1 ± 513.9), 3 doses (179.1 ± 42.7)).

Imaging of dissected organs (liver, lung, spleen, brain, kidney, heart and gonads) demonstrated that protein expression was diminished upon repeat intravenous administration (Fig. 4D). No detectable signal can be visualised in any organ dissected from mice that received 3 doses, in contrast to signal detected in multiple organs from mice treated only once.

Following bioluminescence imaging, organ homogenate was prepared from select organs (lungs, liver and spleen) and luciferase expression quantified in vitro. Figure 4E shows luciferase expression, normalised for total protein, in untransduced mice (UT) and mice that received one or three intravenous doses of rSIV.F/HN vector. A two-way mixed-effects ANOVA revealed significant main effects of organ (*p* < 0.0001) and dose number (*p* < 0.0001), as well as a significant organ and dose number interaction (*p* < 0.01). Post-hoc analysis revealed that mice delivered a single dose of vector had significant (*p* < 0.05 and *p* < 0.001) luciferase expression above untransduced levels in the lungs and spleen, respectively. Mice that received a single vector dose further demonstrated significantly (*p* < 0.05 and *p* < 0.001) higher luciferase expression than repeat-dosed mice in the liver and spleen, respectively. Luciferase expression in mice that received 3 vector doses was not significantly different from untransduced levels in the lung, liver and spleen, suggesting transduction efficiency had been reduced to background levels after repeat rSIV.F/HN intravenous administration.

**Fig. 5:**
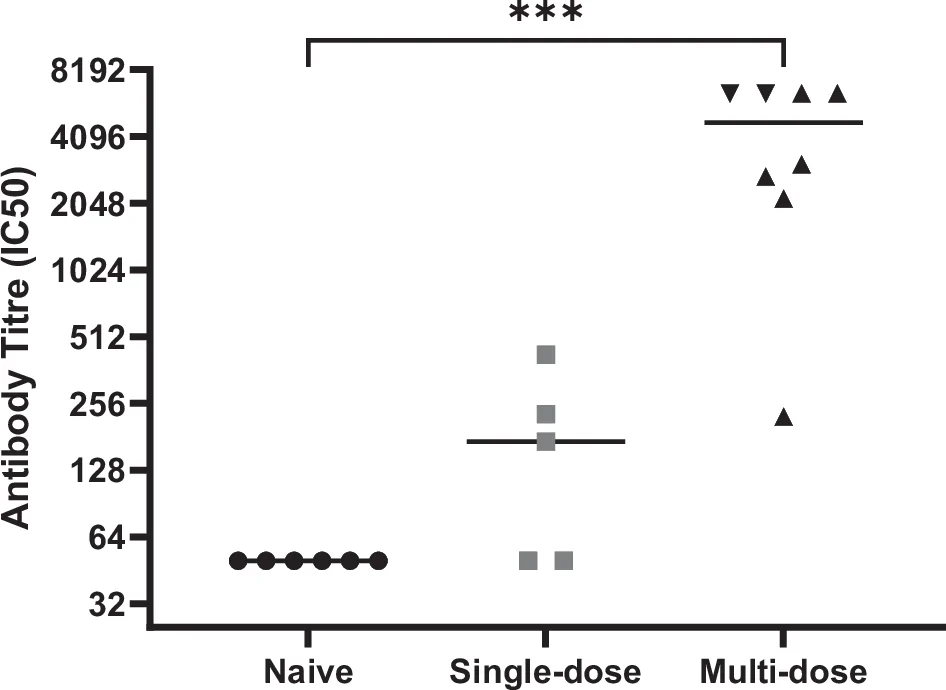
Measurement of neutralising antibodies produced in serum following repeat intravenous doses of rSIV.F/HN. Antibody titre was measured in serum of wildtype C57BL/6 mice that received multiple doses of rSIV.F/HN: one/two doses of control vector (rSIV.F/HN-mADAMTS13, 1.42e8 TU/mouse) and a final dose of rSIV.F/HN-EGFPLux (1.52e8 TU/mouse), and mice that received a single dose of rSIV.F/HN-EGFPLux (1.52e8 TU/mouse). Vector administered at monthly intervals. Blood was sampled from mice one month after each rSIV.F/HN dose, just prior to the intravenous infusion of the next dose. Neutralising antibodies were measured by incubating rSIV.F/HN-EGFP virus with serial dilutions of mouse serum, then measuring the percentage GFP-positive cells by flow cytometry 72 h later. Antibody titre is the inverse of the serum dilution required for 50% virus neutralisation compared to no-serum control (IC50). Neutralising antibody titre shown in untransduced mice (*n* = 6 mice, black circles), mice that received a single dose of rSIV.F/HN (*n* = 5 mice, light grey squares), and multiple doses (two doses *n* = 6 mice dark grey upwards triangles, three doses *n* = 2 mice dark grey downwards triangles). Assay is saturated at 1/6400 serum dilution. Each shape represents a single mouse. Horizontal bars represent the median. *** = *p* < 0.001 comparing untransduced mice and mice receiving a single dose and multiple doses of vector, Kruskal-Wallis with Dunn’s multiple comparison test.

To investigate the generation of neutralising antibodies against F/HN following systemic delivery, serial dilutions of mouse serum were incubated with rSIV.F/HN-EGFP vector, then applied to HEK293T cells. The inverse serum dilution leading to 50% reduction of GFP + cells was taken as the neutralising antibody titre (IC50). Figure 5 shows the neutralising antibody titre measured in mice that received one dose and multiple doses (2 or 3 doses combined) of rSIV.F/HN compared with untransduced mice. After a single intravenous dose, neutralising antibodies against the vector were detectable, although not significantly different from untransduced mice. However, there was a significant (*p* < 0.001) increase in antibody titre with successive administrations when compared to untransduced mice. In some cases, the maximum antibody titre (maximum detection limit of the assay) was reached following two doses, and both mice that received three doses demonstrated maximal neutralising antibody titre.

## Discussion

Intrapulmonary delivery of rSIV.F/HN has been previously characterised, with promising features including persistent pulmonary gene expression (at least 2 years) following a single dose, efficient and highly localised transduction of the respiratory epithelium and feasible repeat administration at daily and monthly intervals without loss of transduction efficacy [5–7]. As a result, administration of rSIV.F/HN has progressed into a first-in-human trial in CF subjects. Importantly, the vector may also hold promise for other disease indications. More recently, rSIV.F/HN delivered topically to murine lungs successfully achieved therapeutically relevant plasma protein levels of the secreted protein Factor VIII [8].

To investigate further the suitability of rSIV.F/HN vector for treatment of systemic diseases, intravenous administration to wildtype mice was investigated and compared with intrapulmonary administration. We show that a single intravenous dose of rSIV.F/HN achieved sustained expression of protein in sera for the duration of the study (12 months). The biodistribution of vector following intravenous infusion was widespread, with significant protein expression measured in the lung, liver and spleen. rSIV.F/HN was repeatedly administered to assess if intravenous vector delivery provokes an anti-vector immune response. A reduction in luciferase expression to background levels was observed in the lung, liver and spleen following three intravenous doses, accompanied by the generation of significant anti-SIV.F/HN NAbs in sera. Together, these data demonstrate that rSIV.F/HN can be administered intravenously to achieve efficient and sustained systemic protein expression (at least 12 months). However, the generation of significant anti-vector NAbs and loss of protein expression show that repeat intravenous administration is not tolerated, and intrapulmonary delivery of rSIV.F/HN should instead be considered if repeat administration is required.

Systemic delivery of lentiviral vectors has historically shown modest efficacy in animal models. Intra-portal delivery of VSV-G FIX lentiviral vectors to dogs with haemophilia B demonstrated long-term reduction in bleeding episodes in treated dogs and up to 1% normal FIX activity [18]. Vector administration was accompanied by acute inflammatory responses and hepatotoxicity that were resolved with prophylactic anti-inflammatory and anti-histamine treatment, an unexpected finding following successful restoration of FIX activity and lack of toxicity in mouse models [18, 19]. Using the same immunosuppression pre-treatment, intravenous administration of VSV-G lentiviral vectors encoding human fumarylacetoacetate hydrolase (FAH) in a pig model of liver metabolic disease, hereditary tyrosinemia type-1 (HT1) led to anaphylactoid reactions associated with elevated innate immune responses, and thus was not well tolerated in pigs [20]. However, when delivered via the portal vein, lentiviral vectors encoding human FAH achieved liver repopulation with FAH-positive hepatocytes in knockout pigs and prevention of precancerous lesions until the end of the study [20]. Although no signs of toxicity were observed in mice that received rSIV.F/HN by intravenous infusion, full characterisation of the innate immune response following vector administration should be performed, particularly in larger animal models, which often do not recapitulate responses in mice. Across the 52-week study, three mice died prematurely in the intrapulmonary group. Two mice were culled based on veterinary advice due to an infected eye wound (7.6e7 TU vector group) and from overgrooming (TSSM group), respectively and one mouse was found dead 36 weeks post-treatment (2.4e7 TU vector group), but the cause was unknown. This mouse was 45 weeks old at the time of death. All deaths were considered incidental rather than treatment-related. This is consistent with our previous study, which demonstrated no evidence of long-term chronic toxicity in mice following intrapulmonary rSIV.F/HN vector administration [6].

In this study, we utilised the human CMV enhancer coupled to the ubiquitous human EF1α short promoter (hCEF), which was previously selected as part of our lead clinical candidate to drive CFTR expression following persistent and efficient gene expression in both the murine nose and lung and human ex vivo air-liquid interface cultures [7]. Following intrapulmonary delivery, it was shown that several cell types within the murine lungs are transduced by the rSIV.F/HN pseudotyped vector, including ciliated epithelial cells, goblet and club cells and type I and II pneumocytes [7]. In human ALI cultures, a similar range of cell types was transduced by rSIV.F/HN, which is not unexpected given Sendai virus envelope protein HN binds to sialylated glycans, specifically α2,3 sialylated N-acetyllactosamine (LacNAc), which is prevalent on human airway cells [21].

The tropism of rSIV.F/HN when delivered systemically should be dictated by the expression of Sendai virus HN receptors, known to be ubiquitous across many cell and tissue types [21]. Successful transduction has been demonstrated with recombinant Sendai vectors in the vascular endothelium, skeletal muscle, cardiomyocytes, upper and lower respiratory tract, brain and retina across multiple species, including rats, mice, ferrets and sheep [22–28]. The results presented here from the firefly luciferase biodistribution study revealed that systemic delivery of rSIV.F/HN vector results in widespread organ transduction, reflecting previous literature utilising recombinant Sendai vectors. A limitation of intraperitoneal luciferin injection is that not all organs take up the substrate equally, potentially underestimating luciferase expression and vector biodistribution to some organs. To mitigate this limitation, luciferase expression was further quantified in organ homogenate to more accurately assess vector biodistribution. To improve the efficiency of systemically delivered lentiviral vectors and to prevent expression in off-target organs, many have investigated the use of tissue-specific promoters to drive transgene expression, for instance, the use of the alpha-1-antitrypsin (AAT) promoter to drive hepatic expression [20]. Although no specific therapeutic application was investigated here, inclusion of a liver-specific promoter could be assessed in the future to drive hepatic rSIV.F/HN transgene expression for clotting disorders and other similar indications.

Incorporation of an inhibitor of phagocytosis, CD47, in lentiviral vector producer cells was shown to mitigate VSV-G vector clearance by liver phagocytes, reduce recognition by the innate immune system, and enhance hepatocyte gene transfer and FIX activity in non-human primates [29]. Phagocytosis of lentiviral vectors by antigen-presenting cells (APCs) in the liver and spleen therefore, highlights an impediment to systemic vector delivery [30]. Considering significant transduction of these organs is observed following systemic rSIV.F/HN delivery, incorporation of CD47 might be beneficial in the future. Detection of low levels of bioluminescence in the gonads of some mice following intravenous rSIV.F/HN delivery raises an important safety concern and potential impediment to systemic vector delivery due to the risk of lentiviral genome insertion into germ cells and germline transmission. Although bioluminescence was not significantly above background levels in treated mice, it should be established if lentiviral genome can be detected in germ cells following systemic rSIV.F/HN delivery. However, this is unexpected as lentiviral vectors pseudotyped with both Sendai virus F and HN envelope proteins were unable to transduce germline stem cells when cultured ex vivo [31]. Clearly, an advantage of intrapulmonary vector delivery is the containment of vector in the thoracic cavity, shown here by the lack of bioluminescence detected in murine gonads following intrapulmonary rSIV.F/HN delivery.

Vector biodistribution was assessed using lentivirus encoding firefly luciferase and EGFP fusion (rSIV.F/HN-EGFPLux) by luciferase quantification in live mice and organ homogenate and EGFP detection by direct fluorescence microscopy of organ slices to determine cell-type expression. Analysis of liver and spleen tissue slices from mice delivered rSIV.F/HN-EGFPLux by intravenous administration did not reveal any detectable EGFP signal. This is likely due to an overall low transduction efficiency, which makes it difficult to find individual transduced cells in a large organ such as the liver, or quenching of the GFP fluorescence due to fusion with luciferase  (data not shown). Cell-type-specific determination of rSIV.F/HN transduction could instead be investigated in the liver and spleen using an EGFP vector and flow cytometry. Alternatively, vector biodistribution could be assessed by RNA extraction from mouse tissue and determination of vector genomes by quantitative PCR. Unfortunately, this was beyond the scope of the current study due to technical limitations. EGFPLux was also selected for repeat administration studies to provide a simple method to compare gene expression between mice that received one and three rSIV.F/HN doses. Control rSIV.F/HN vector encoding mADAMTS13 was selected as an irrelevant vector because we previously concluded that matching doses of rSIV.F/HN mADAMTS13 vector delivered intravenously to mice did not achieve detectable levels of protein (data not shown), and thus was not predicted to influence the phenotype of wildtype mouse.

The long-term duration study demonstrated that rSIV.F/HN administered via either the intrapulmonary or intravenous routes leads to stable transgene expression in serum for the duration of the experiment (at least 12 months). This supports previous reports whereby a single intrapulmonary dose of rSIV.F/HN-GLux demonstrated stable expression in sera twelve months post-transduction [8]. The overall trend in both the intravenous and intrapulmonary cohorts was a dose-related increase in transduction efficiency, with dose determined as a significant factor influencing serum expression. The delivery method with superior generation of reporter protein was dependent on the vector dose, as shown by a significant interaction of dose and delivery route. Intrapulmonary delivery was more potent at the lowest dose (2.4e7 TU/mouse), both delivery methods performed equally at the medium dose, and intravenous delivery demonstrated enhanced efficacy (approximately 10-fold) at the highest dose tested (2.4e8 TU/mouse). Vector delivered by intravenous administration demonstrated a threshold dose below which GLux expression was at background levels. This may be due to the vector being diluted among multiple organs following systemic delivery compared with the highly localised transduction of the nose and lungs following intrapulmonary delivery. This study suggests that higher doses of rSIV.F/HN are required to achieve detectable transgene expression in serum following intravenous than intrapulmonary delivery.

Following demonstration of sustained long-term expression following a single dose, rSIV.F/HN was repeatedly administered, whereby luciferase expression in mice that received 3 vector doses was at background levels in the lung, liver and spleen. Here, luciferase expression was quantified at the terminal time-point only, as only the final vector encoded luciferase. However, neutralising antibody titre was monitored longitudinally as sera were sampled from mice after one, two or three vector administrations. Mice that received a single intravenous dose demonstrated a detectable level of neutralising antibodies in sera, and after two doses, a strong induction of neutralising antibodies was observed, which plateaued to the maximum antibody titre following three doses, albeit with limited *n* numbers. We cannot exclude that cellular immune responses induced after the second and third dose lead to the elimination of transduced cells. However, the experiment was not designed to study cellular immune responses, and instead, we focused on tissue luciferase expression and anti-vector responses with repeat intravenous administration. In contrast, we have shown previously that sustained transduction efficiency is demonstrated following repeat intrapulmonary administration of rSIV.F/HN [6]. One difference in experimental design was the dose delivered to mice (1e7 TU/mouse intrapulmonary, 1.5e8 TU/mouse intravenously), with the 15-fold difference potentially eliciting a stronger immune response due to a greater number of vector particles. It is plausible that a lower dose of vector might be tolerated following repeat intravenous delivery. However, this seems unlikely given that a moderate neutralising antibody response was generated following just one intravenous administration. Rhesus monkeys delivered two intravenous doses of lentiviral vector, noted substantial anti-vector responses after one dose, which completely inactivated the second lentiviral administration [32].

In summary, rSIV.F/HN lentiviral vectors successfully achieved persistent and widespread expression of a reporter protein following a single intravenous dose. Broad tropism may suggest the potential of rSIV.F/HN vector as a therapeutic for wider disease indications. For example, respiratory diseases of the pulmonary endothelium, haematological diseases including clotting disorders, infectious diseases and inherited metabolic disorders, amongst others. The effective transduction of organs and tissues other than the lungs may allow greater flexibility in the selection of transgene, including those which encode proteins that are more efficiently post-translationally modified in organs such as the liver rather than the airways.

The production of a strong anti-vector response following repeat intravenous administration may suggest that systemic rSIV.F/HN delivery is not suited for chronic diseases that require lifelong transgene expression and repeat dosing. However, other therapeutic viral vectors, such as AAV, are equally limited to single administrations due to immune recognition, yet have shown remarkable long-term correction of disease following a single AAV dose [12]. Moving forward, strategies such as tissue-specific promoters could be investigated to enhance transgene expression in desired organs. Alternatively, utilising the lungs as a factory for production of systemic proteins has demonstrated sustained expression of therapeutic transgenes in serum with feasible repeat administration. Intrapulmonary administration might therefore be an efficient yet safer delivery method than systemic delivery moving forward, particularly if the disease application requires long-term gene delivery.

## Supplementary information

Supplementary Figure

## Data Availability

Additional data are available from the corresponding author upon reasonable request.
