# Supplementary material for: Assessment of F/HN-pseudotyped lentiviral vector following intravenous delivery to mice
Source: Gene Ther. 2026 Jun 18;33(5):586–95. doi: 10.1038/s41434-026-00623-3 (PMC13585553; doi:10.1038/s41434-026-00623-3)
Supplement: Supplementary file 1 [file 41434_2026_623_MOESM1_ESM.pdf]

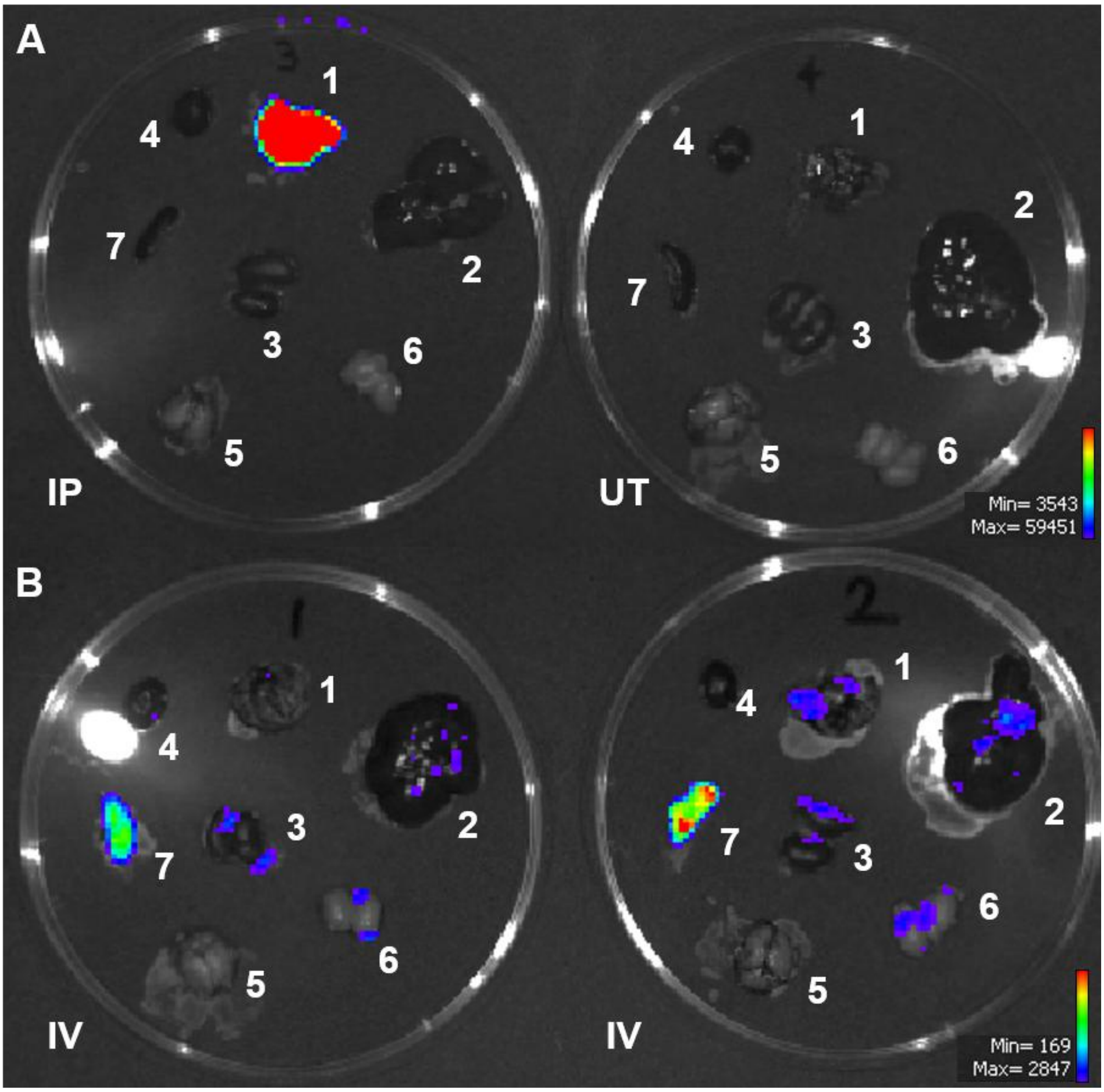

Supplementary Figure 1 - Bioluminescence imaging of organs dissected from mice transduced with rSIV.F/HN-EGFPLux.

Vector encoding the firefly luciferase and EGFP fusion (rSIV.F/HN-EGFPLux) was delivered to C57BL/6 wildtype mice by intrapulmonary (IP n=3 females and n=3 males, 2.58e8 TU/mouse) and intravenous administration (IV n=4 females and n=4 males, 2.58e8 TU/mouse) alongside untransduced mice (n=3 females and n=2 males). 35-days post-transduction, each mouse received 150 mg/kg of luciferin substrate by intraperitoneal injection, and approximately 30-minutes later (1) lung (2) liver (3) kidney (4) heart (5) brain (6) gonads and (7) spleen dissected and imaged using the IVIS Spectrum CT. Representative images of (A) a male mouse dosed via intrapulmonary route (IP) and a male untransduced mouse (UT) and (B) two male mice delivered vector intravenously (IV). 5-minute exposure per image. Min and Max represent the minimum and maximum signal, measured in counts, per image.
